# Impact of COVID-19 Pandemic on Gynecologic Oncology Surgery in Brasil

**DOI:** 10.1055/s-0041-1741121

**Published:** 2021-12-06

**Authors:** Francisco José Candido dos Reis

**Affiliations:** 1 Faculdade de Medicina de Ribeirão Preto, Universidade de São Paulo, Ribeirão Preto, SP, Brasil

The COVID-19 pandemic has influenced every aspect of human life for the past 20 months. To date, we have registered more than 21,500,000 cases of the disease and more than 610,000 deaths in Brazil. There were two waves of the disease, one peak in June 2020 and another in March 2021. The entire Brazilian health system has suffered a significant impact from the pandemic. States and cities carried out the National Brazilian Health System (SUS) response to the pandemic without national coordination.

The first measures aimed to reduce the virus dissemination and to protect individuals with high risk of complications. The rapid advance of the pandemic highlighted the insufficiency of intensive care beds, personal protective equipment, and human resources to face the crisis. We observed then the massive and indiscriminate closing of health services dedicated to the treat other diseases. Initial evidence suggested that patients contaminated with covid 19 after the surgical procedure had high mortality. 1 Non-urgent surgeries were postponed and treatment protocols modified.

The reduction in the number of elective and oncologic surgeries has occurred worldwide. Global estimates suggest that about 38% of cancer surgeries were not performed on time. In Brazil, 43% of cancer surgeries have been canceled or postponed. 2 With the perception that the pandemic would be long-lasting, several countries developed strategies to resume the lines of care for cancer patients. An important strategy was the collaboration between services under the coordination of health authorities to implement COVID-19-free hospitals to carry out surgical treatments, including cancer surgeries. 3 Unfortunately, the lack of effective central coordination did not allow the implementation of strategies like this on a large scale in our country. Data collected up to March 2021 showed a dramatic reduction of surgeries in April and May 2020 in Brazil. There has been a slow recovery since then, but without reaching the rates practiced between 2016 and 2019. The backlog is above 1,000,000 surgeries. 4

The delay or cancellation of surgeries of gynecological cancer has several negative impacts. The harm to patients is unquestionable. Poor access to specialized care and delay in surgical treatment are important prognostic factors in endometrial cancer. The mortality of patients with low-risk endometrial cancer is significantly higher than baseline when surgery is performed eight weeks after the diagnosis and worsens as the time to surgery increases. 5 In ovarian cancer, the quality of primary surgery is an independent prognostic factor. Women who have no residual disease at the primary debulking have more prolonged disease-free survival and overall survival than women with residual disease. Ovarian cancer screening trials for early diagnosis have failed to show survival benefits. But, the prompt intervention in patients with suspected ovarian tumors may increase the rate of complete debulking. Delaying the primary surgery can increase the risk of complications like bowel obstruction and respiratory insufficiency and limit treatment options. Cancer of the cervix is a significant problem in Brazil. More than 16.000 cases are diagnosed each year, and the majority are in the advanced stage. There is evidence that the delay in surgery can increase the parametrial invasion rates and the need for adjuvant treatment. Longer wait-time for radiotherapy is also associated with an increased risk of mortality.

The COVID-19 pandemic has also negatively impacted surgical training in gynecologic oncology. A recent study reported a worse overall impact for trainees in countries with no national training program. 6 Countries with organized national training systems seem more effective in providing faster solutions for the safe resumption of surgical procedures, minimizing losses in learning opportunities.

Several measures are needed to mitigate the negative effects of the COVID-19 pandemic on gynecological cancer care. We need high-quality studies to provide accurate data on the backlog of patients to plan a rational allocation of resources. Additional resources must be provided to reference hospitals to increase treatment capacity for patients on waiting lists and the likely increase of patients with invasive tumors secondary to screening interruptions. Training programs must carefully assess the need to replace learning activities not carried out during the pandemic.

The COVID 19 pandemic has exposed several weaknesses in the care of women with gynecological malignancies. We must learn the lessons of this challenging period. We need a coordinated health system that responds quickly to public health emergencies without neglecting the care of other diseases such as malignant neoplasms. We also need a robust national training structure in gynecology oncology, essential to maintaining effective patient care and training in difficult times.
